# Calcium Deficiency Rickets in African Adolescents: Cortical Bone Histomorphometry

**DOI:** 10.1002/jbm4.10169

**Published:** 2019-02-11

**Authors:** Christine M Schnitzler, John M Pettifor

**Affiliations:** ^1^ MRC/Wits Developmental Pathways for Health Research Unit Department of Paediatrics University of the Witwatersrand Johannesburg South Africa

**Keywords:** RICKETS, CALCIUM DEFICIENCY, ADOLESCENCE, OSTEOMALACIA, CORTICAL BONE HISTOMORPHOMETRY

## Abstract

Rickets due to dietary calcium deficiency has been well described in black African children, but less is known about this condition in black adolescents. We investigated 26 black adolescents (19 males aged 11 to 19 years and 7 females aged 12 to 15 years) with rachitic leg deformities and 20 controls by routine iliac crest undecalcified cortical bone histomorphometry for disturbances of bone turnover and for mineralization defects, including severity of osteocytic osteolysis (Ot.Olysis) and periosteocytic osteolysis (Peri.Ot.Olysis) of the lacunar‐canalicular space. Serum levels of calcium (sCa), 25‐hydroxyvitamin D (25OHD), 1,25‐dihydroxyvitamin D (1,25(OH)_2_D), and total alkaline phosphatase (ALP) were measured. Histomorphometry showed varying degrees of severity of secondary hyperparathyroidism (2^0^ HPT) characterized by hyperosteoidosis, increased erosion, and porosis. Because osteoid was neither being mineralized nor eroded (osteoclasts cannot erode osteoid), it increasingly blocked bone surface needed for osteoclastic resorption. Where osteoid covered >50% of bone surface, osteoid thickness, severity of Ot.Olysis, and extent of Peri.Ot.Olysis increased, sCa and 25OHD declined, and 1,25(OH)_2_D and ALP increased. At 80% osteoid cover, bone remodeling had all but ceased, secondary HPT had changed to osteomalacia, and serum biochemical results had deteriorated further. Disease severity was greater in males than in females, likely because males grow faster and for longer than females. In conclusion, this cross‐sectional clinical case study presents cortical bone histomorphometric data of secondary HPT and its transition to osteomalacia in black adolescents with rickets attributable to dietary calcium deficiency. The bone disease was most severe in older adolescent males. Importantly, bone pathology of calcium deficiency rickets in adolescents was not confined to bone surfaces but also manifested at osteocyte level as Ot.Olysis and Peri.Ot.Olysis. © 2019 The Authors. *JBMR Plus* published by Wiley Periodicals, Inc. on behalf of American Society for Bone and Mineral Research.

## Introduction

Rickets is a metabolic bone disease of deficient mineralization resulting in deformities of growing bone that has been reported from at least 59 countries.^1^, ^2^ Whereas vitamin D deficiency is a cause of rickets in infants and children in the first 2 years of life the world over, dietary calcium deficiency as a cause of rickets in children and adolescents aged 2 to 15 years has been reported predominantly from low‐ and middle‐income countries in Asia and Africa.^1^ In most of Africa, calcium deficiency is attributable to low calcium content of the staple diet consisting mainly of grain and vegetables but few or no dairy products.^3^ A recent randomized controlled trial established that a daily amount of at least 1000 mg of oral calcium supplementation for 6 months or longer is required for radiographic bone healing of dietary calcium deficiency rickets in children.^4^ The therapeutic response is improved with additional vitamin D therapy.^5^

Dietary calcium intake by rural black South African adolescents has been reported to be as low as 229 mg per day,^3^ an amount well below the recommended daily allowance for 6‐month‐old North American infants and that for adolescents of 1300 mg.^6^

Pathological changes of calcium deficiency rickets manifest in both cartilage and bone. In cartilage, histopathological changes in the metaphyseal growth plate are failure of mineralization of the cartilaginous septae between vertical columns of hypertrophic growth cartilage cells. When subjected to weight‐bearing, the cartilaginous septae and intervening cartilage cell columns collapse into disorganized tissue that becomes the site of deformity.^7^ Histopathological changes of cortical bone in calcium deficiency are mineralization failure of cortical bone surfaces and demineralization of mineralized bone tissue, a process referred to as osteocytic osteolysis. In this process, osteocytes enlarge both their lacuna and canaliculi by demineralization but demineralized osteoid remains in place. If this osteoid is also removed, some speak of “periosteocytic osteolysis.” Bone resorption by multinucleate osteoclasts refers to the physiological process of removal of old bone to allow continuing bone renewal. Excessive numbers of osteoclasts lead to bone loss.

In calcium deficiency rickets during childhood, weight‐bearing bones develop deformities,^1^ but general well‐being is usually not affected and pain is rarely a feature. Whereas calcium deficiency rickets in childhood has been well described,^1^, ^8^ its presentation during adolescence, particularly in older adolescents, has received little attention. Available bone histomorphometric data in rickets are limited to trabecular bone.^9^, ^10^ In our previous study of the trabecular abnormalities in the same group of adolescents who were studied in the present study, we found that all had bone disease with 46% having osteopenia with normal or low bone turnover, 19% having mildly increased bone turnover, 15% histologic hyperparathyroidism, 8% pre‐osteomalacia, and 12% osteomalacia (with features of hyperparathyroidism).^10^ The present study is the first to examine cortical bone for histomorphometric abnormalities in dietary calcium deficiency in adolescents aged 11to 19 years.

## Subjects and Methods

### Patients

**Figure 1:**
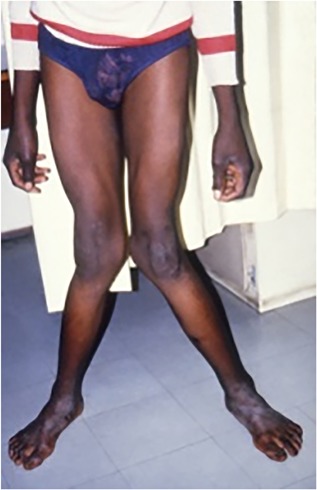
A 17‐year‐old patient with marked genu valgum deformities due to dietary calcium deficiency.

This is a cross‐sectional study of 26 black adolescents, 19 males aged 11 to 19 years (15 of these were aged 16 to 19 years) and 7 females aged 12 to 15 years (Table 1) who complained of angular knee deformities (genu valgum or genu varum deformities or both) (Fig. 1) of spontaneous onset between 1 and 15 years ago. There was no radiographic evidence of injury at the deformed site. The patients were investigated for metabolic bone disease and an iliac crest bone biopsy (after tetracycline double bone labeling) had been obtained at the time of corrective surgery between 1987 and 1990. Trabecular bone histomorphometry data and serum biochemical abnormalities had shown a range of abnormalities consistent with calcium deficiency, namely osteoporosis, hyperparathyroid bone disease, pre‐osteomalacia, and osteomalacia.^10^ Cortical bone biopsy results from the same specimens were obtained recently and are reported here, together with the biochemical data obtained at the time of the bone biopsy. Cortical tetracycline‐based data are omitted here because of the time lapse since tetracycline labeling.

**Table 1:** Age and Sex Distribution of 26 Patients and 20 Control Subjects

	Patients (*n* = 26)				Controls (*n* = 20)			
Age (years),^a^ mean ± SD (range)	15.5 ± 2.4 11–19				14.2 ± 3.3 (9–20)			
	Males^b^ (*n* = 19)		Females (*n* = 7)		Males (*n* = 14)		Females (*n* = 6)	
Age groups (years)	*n*	Age	*n*	Age	*n*	Age	*n*	Age
9–10	0		0		3	9.7 ± 0.6	0	
11–12	2	11.5 ± 0.7	2	12.0	3	11.3 ± 0.6	1	12.0
13–14	1	13.0	3	13.3 ± 0.6	5	13.6 ± 0.5	0	
15–16	7	15.9 ± 0.4	2	15.0 ± 0	1	16.0	2	15.5 ± 0.7
17–18	5	17.4 ± 0.5	0		1	17.0	1	17.0
19–20	4	19.0 ± 0	0		1	20.0	2	19.5 ± 0.7
Age (years),^a^ mean ± SD (range)	16.3 ± 2.3 (11–19)		13.4 ± 1.3 (12–15)		13.1 ± 3 (9–20)		16.5 ± 2.9 (12–20)	

^a^ Age of patients versus controls, *p* = 0.128; age of males versus females among patients, *p* = 0.01, and among controls, *p* = 0.057.

^b^ Sex distribution between patients and controls, *p* = 0.818.

At the time of biopsy, the patients’ nutritional status was not well defined but was reported to be adequate on the customary staple food consisting of maize (corn), vegetables, and some meat. Itemized dietary questionnaires were not administered because of communication problems relating to customary diets, particularly in patients from rural areas. Consumption of dairy products had been minimal (milk in tea) or absent. The duration of the leg deformity was vague and had been given as between 1 and 15 years. The principal complaint was an awkward gait, but pain and muscle weakness had been absent. Genu valgum (knock knees) was more common than genu varum (bow legs), and most deformities were bilateral and severe with intermalleolar distance of 20 cm or more in cases of genu valgum. At the time of admission, none of the children were placed on calcium or vitamin supplements before biopsy, although all received a normal ward diet, the calcium content of which was appropriate for adolescents. The study was approved by the Committee for Research in Human Subjects of the University of the Witwatersrand, Johannesburg, South Africa, and informed consent was obtained from the patients’ guardians.

Control values for age‐related cortical histomorphometric variables were obtained from iliac crest bone samples of 20 black subjects (Table 1). Sixteen of these samples were taken at autopsy of previously healthy black individuals who had died a sudden, often violent death and who had no demonstrable organic disease at autopsy. Four samples were obtained from otherwise healthy black patients during orthopedic surgical procedures.

### Iliac crest bone biopsies

Cylindrical iliac crest bone samples, 7.5 mm in diameter, were taken with a Mayo bone biopsy trephine (Zimmer USA, Warsaw, IN, USA) from the standard site 2 cm below the iliac crest and 2 cm behind the anterior superior iliac spine.^11^ Undecalcified methylmethacrylate‐embedded bone cylinders were cut at 7 μm thickness on a Jung K heavy‐duty microtome, and from each specimen three sections, consisting of both cortices and intervening trabecular bone, were stained with Goldner trichrome stain. Measurements were carried out manually by point and intersect counting, or linear measurement using a Zeiss microscope fitted with a Zeiss Integrations Platte II (100 points, 10 lines) in one eyepiece and a graticule (ruler) in the other. Both eyepiece inserts were calibrated with ×3.2, ×10, ×25, and ×40 objectives. The nomenclature of all measured and calculated variables is that approved by the American Society of Bone and Mineral Research 2013.^12^ The three‐dimensional notation was used. Data of measured and calculated variables are listed in Tables 2, 4, and 5

**Table 2:** Structural and Static Histomorphometric Variables in the External Cortex of Iliac Crest Bone Samples of Patients and Controls by Sex

	Boys					Girls						
	Patients		Controls			Patients		Controls				
	*n*	Mean ± SD	*n*	Mean ± SD	*p* Value	*n*	Mean ± SD	*n*	Mean ± SD	*p* Value	B vs. G patients *p* value	B vs. G controls *p* value
Structure												
Ct.Th µm	18	1170 ± 645	14	950 ± 400	0.464	7	1195 ± 509	6	786 ± 127	0.16	0.72	0.492
Md.V/BV %	19	80.7 ± 12.9	14	89 ± 8	0.018	7	89 ± 4	6	89 ± 4	1.0	0.106	0.598
Ct.Vd.V/BV %	19	15.1 ± 11.8	14	9.7 ± 7.8	0.065	7	8.4 ± 3.4	6	8.9 ± 3.2	0.626	0.161	0.544
Ct.OV/BV %	19	4.24 ± 4.13	14	1.6 ± 1.2	0.018	7	2.2 ± 1	6	1.6 ± 0.7	0.305	0.335	0.655
Peri.Ot.Olysis.V/Md.BV %	18	0.3 ± 0.54	14	0	0.047	7	0.05 ± 0.14	6	0	0.455	0.405	1.0
Static bone turnover												
Intracortical envelope												
Ct.OS/BS %	19	49.3 ± 18.9	14	27 ± 12	0.003	7	41 ± 10	6	25 ± 14	0.099	0.178	0.492
Ct.O.Th µm	19	18.1 ± 9.9	14	12 ± 5	0.026	7	13 ± 4	6	14 ± 4	0.727	0.283	0.776
Ct.ES/BS %	19	25.5 ± 14.7	14	20 ± 9	0.231	7	20 ± 6	6	22 ± 15	0.944	0.186	0.968
Ct.ES/(BS–OS) %	19	53.4 ± 28.4	14	29.9 ± 16.4	0.03	7	33.6 ± 10.4	6	30 ± 21.1	0.532	0.061	0.968
Endocortical envelope												
Ec.OS/BS %	19	41.8 ± 24.5	14	18 ± 13	0.007	7	30 ± 13	6	17 ± 10	0.099	0.236	0.776
Ec.O.Th µm	19	16 ± 8.6	13	13 ± 7	0.384	7	15.5 ± 5.9	6	11 ± 3.2	0.248	0.909	0.635
Ec.ES/BS %	19	28 ± 18.4	14	19 ± 17	0.14	7	23 ± 20	6	16 ± 11	0.626	0.864	0.935
Ec.ES/(BS–OS) %	19	54.7 ± 30.8	14	22.9 ± 20.1	0.008	7	33.4 ± 29.9	6	21.6 ± 16.9	0.626	0.169	0.776
Periosteal envelope												
Ps.OS/BS %	18	83 ± 29	13	41 ± 34	0.009	6	58 ± 36	6	24 ± 15	0.092	0.232	0.415
Ps.O.Th µm	18	13.8 ± 7.9	11	7.3 ± 2.1	0.027	6	8.8 ± 3.5	6	5.7 ± 1	0.201	0.207	0.194
Ps.ES/BS %	18	8.36 ± 17.8	13	7.2 ± 9.4	0.165	6	17 ± 15	6	16 ± 9	0.584	0.123	0.13
Ps.ES/(BS–OS) %	9	46.7 ± 48.3	11	12 ± 12.4	0.391	4	44.4 ± 19.9	6	22.9 ± 13	0.144	0.819	0.21

B vs. G = boys vs. girls; Ct.Th = cortical thickness; Md. V/BV = mineralized bone volume/bone vol; Ct.Vd.V/BV = cortical void vol/bone vol; Ct.OV/BV = cortical osteoid vol/bone vol; Peri.Ot.Olysis.V/Md.BV = periosteocytic osteolysis vol/mineralized bone vol; Ct.OS/BS = cortical osteoid surface/bone surface; Ct.O.Th = cortical osteoid thickness; Ct.ES/BS = cortical eroded surf/bone surf; Ct.ES/(BS–OS) = cortical eroded mineralized bone surf; Ec.OS/BS = endocortical osteoid surf/bone surf; Ec.O.Th = endocort osteoid thickness; Ec.ES/BS = endocort eroded surf/bone surf; Ec.ES/(BS–OS) = endocort eroded mineralized bone surf; Ps.OS/BS = periosteal osteoid surf/bone surf; Ps.O.Th = periost osteoid thickness; PsES/BS = periost eroded surf/bone surf; Ps.ES/(BS–OS) = periost eroded mineralized bone surf.

Data of the external iliac crest cortex were chosen for analysis in all samples because modeling of growth at the external iliac crest cortex reflects that of long bone diaphyses as both sites undergo periosteal expansion and endocortical resorption and show marked porosity during growth.^13^

### Histomorphometric analysis—patients and controls

Bone surface variables were identified by their envelope‐specific prefixes Ct (short for intracortical), Ec (endocortical), and Ps (periosteal), eg, Ct.OS/BS % for intracortical osteoid surface % of total intracortical bone surface. Bone surface activities are envelope‐specific. The cortical envelope is characterized by remodeling, which replaces old bone with new by sequential resorption coupled with subsequent formation at the same site. At the other two envelopes, modeling achieves bone enlargement and preservation of proportions of shape by simultaneous bone resorption and formation at different sites: During adolescence, the endocortical envelope undergoes modeling for intermittent net bone loss or gain, and the periosteal envelope modeling for net bone gain.^14^

### Structural and static bone turnover variables (Table  2)

Cortical thickness (Ct.Th, μm) was measured orthogonally to the periosteal surface at 8 equidistant intervals (magnification ×32). Cortical bone volume (BV %) was measured by point counting (mag × 100) of the total cortical bone tissue area. Total points were considered to be bone volume BV % = 100%. Mineralized bone volume was calculated as Md.V % = 100 % BV − (Vd.V % + OV %). Void volume (Vd.V/BV %) and osteoid volume (OV/BV %) were each measured by point counting (mag ×100) and were expressed as percentage of bone volume. Osteoid surface (OS/BS %) and eroded surface (ES/BS %) were measured by intercept counting (mag ×100) and expressed as percentage of total surface intercepts. Eroded mineralized surface (ES/(BS–OS) %) was calculated to eliminate osteiod surface from the denominator because osteoid cannot be eroded by osteoclasts and is therefore meaningless in bone turnover calculations. Osteoid thickness (O.Th, μm) was measured at about 80 μm intervals (mag ×250) using the graticule.

### Features of demineralization of cortical bone tissue

In rickets, bone mineral is removed in two ways: 1) In bone surface erosion, osteoclasts remove both bone mineral and osteoid; 2) In both osteocytic osteolysis^15^ (Ot.Olysis) and in periosteocytic osteolysis (Peri.Ot.Olysis), osteocytes remove bone mineral from the lacunar‐canalicular space and from perilacunar bone (Peri.Ot.Olysis); however, demineralized osteoid remains in place.

Whereas osteoclastic surface erosion is accessible to investigation by most standard laboratory microscopes, study of osteocytic osteolysis requires higher power of resolution.

In the absence of such appropriate imaging technology, we used visual scanning of the external cortex of one Goldner‐stained section of each specimen under ordinary light microscopy at mag ×400 for the presence of qualitative and semiquantitative changes described as grades 2 to 4 of osteocytic osteolysis (Ot.Olysis) and the perilacunar area (Peri.Ot.Olysis) in Table 3.

**Table 3:** Histological Grades of Osteocytic Osteolysis (Ot.Olysis)

Variable	Grade 1^a^	Grade 2^b^	Grade 3^b^	Grade 4^b^
Lacuna	Small, oblong	**Enlarged, round**	**Enlarged, round**	**As Grade 3**
Lacunar content	Unstained	**Pink**	**Dark red**	**As Grade 3**
Osteocyte	Small, dark	**Enlarged, dark**	**Enlarged, dark**	**As Grade 3**
Canaliculi	Invisible	Invisible	**Dark, tortuous, directed to other osteocytes or to an osteoid surface**	**As Grade 3**
Perilacunar area	Normal bone	Normal bone	Normal bone	**Bright red, variable size**

^a^ Grade 1 = normal.

^b^ Abnormal grades in bold.

In addition, each volume of Peri.Ot.Olysis was measured in two orthogonal directions using the eyepiece graticule at mag ×100 (recorded in μm^3^), and expressed this as percentage of mineralized bone volume Md.V/BV %. A distinction between Ot.Olysis and Peri.Ot.Olysis was necessary for purposes of quantitation because both features could not be measured by the same method because 1) features of Ot.Olysis are dimensionless, and 2) in order to establish correlations with histomorphometric and biochemical variables.

### Serum biochemistry

Serum creatinine was measured in all patients. Serum levels of calcium (sCa) and alkaline phosphatase (ALP) were measured in 19 patients, 25OHD in 18, and 1,25(OH)_2_D in 16 patients. Parathyroid hormone was not measured (cost). Details of laboratory methods and results of sCa, 25OHD and 1,25(OH)_2_D were reported previously.^(10)^ Control values for biochemical variables were taken from normal laboratory reference values.

### Statistical analysis

Statistical analysis was carried out using the Statistical Analysis System SAS 9.1 (SAS Institute, Cary, NC, USA). Sex distribution of patients and controls was tested by the chi‐square test (Table 1). The Wilcoxon rank‐sum test was used for comparisons in Tables 1 and 2. Correlations were expressed by the Spearman rank correlation coefficients in Tables 4 and 5

**Table 4:** Correlations Between Bone Histomorphometric and Serum Biochemical Variables in Patients

	Serum biochemistry		
Histomorphometry	sCa (mmol/L)	25OHD (nmol/L)	ALP (U/L)
Md.V/BV %	0.472^a^	0.482	−0.062
	**0.042** ^b^	**0.043**	**0.002**
Ct.Vd.V/BV %			0.677
			**0.001**
Ct.OV/BV %	−0.607	−0.556	0.617
	**0.006**	**0.017**	**0.005**
Ct.OS/BS %	−0.598	−0.552	0.455
	**0.007**	**0.018**	**0.051**
Ct.O.Th μm	−0.693	−0.536	0.6
	**0.001**	**0.022**	**0.011**
Ec.O.Th μm		−0.509	
		**0.031**	
Ps.O.Th μm			0.561
			**0.018**
Ot.Olysis grade	−0.666	−0.454	
	**0.002**	0.059	
Peri.Ot.Olysis.V/Md.BV %	−0.769	−0.558	
	**0.0002**	**0.02**	
Ct.ES/(BS–OS) %		−0.571	0.669
		**0.013**	**0.002**
25OHD nmol/L			−0.544
			**0.02**
1,25(OH)_2_vit D pmol/L		−0.491	0.621
		0.053	**0.01**

Md.V/BV = mineralized volume/bone vol; Ct.Vd.V/BV = cortical void vol/bone vol; Ct.OV/BV = cort osteoid vol/bone vol; Ct.OS/BS = cort osteoid surface/bone surf; Ct.O.Th = cort ost thickness; Ec.O.Th = endocortical ost thickness; Ps.OTh = periosteal ost. thickness; Ot.Olysis = osteocytic osteolysis; Peri.Ot.Olysis.V/Md.BV = periosteocytic osteolysis vol/Md.BV; Ct.ES/(BS–OS) = cort eroded mineralized surf.

^a^ *ρ*, Spearman rank correlation coefficient.

^b^ *p* value.

**Table 5:** Correlations of Osteocytic Osteolysis (Ot.Olysis) and Periosteocytic Osteolysis (Peri.Ot.Olysis) Versus Biochemical and Histomorphometric Variables in Patients as Markers of Calcium Deficiency (Serum Calcium Was the Most Powerful Marker of the Severity of Calcium Deficiency)

	*n*	*ρ* ^a^	*p* Value
Osteocytic osteolysis grades			
sCa mmol/L	19	−0.666	**0.002**
Peri.Ot.Olysis.V/Md.BV %	25	0.536	**0.006**
Ct.OV/BV %	26	0.41	**0.04**
Ct.OS/BS %	26	0.408	**0.039**
Periosteocytic osteolysis volume/Md.bone volume %			
sCal mmol/L	18	−0.777	**0.0002**
25OH D nmol/L	17	−0.558	**0.02**
Md.V/BV%	25	−0.527	**0.007**
Ct.OV/BV%	25	0.65	**0.0004**
Ct.OS/BS%	25	0.471	**0.017**
Ct.O.Th μm	25	0.559	**0.004**
Ec.O.Th μm	25	0.476	**0.016**
Ps.O.Th μm	24	0.542	**0.006**
Ec.ES/(BS–OS)%	25	0.614	**0.001**

sCalcium = serum calcium; Peri.Ot.Olysis.V/Md.BV = periosteocytic osteolysis vol/mineralized BV; Ct.OV/BV = cortical osteoid vol/bone vol; Ct.OS/BS = cort ost surface/bone surface; 25OH D = 25‐hydroxyvitamin D; Md.V/BV = mineralized volume/bone vol; Ct.O.Th = cort ost thickness; Ec.O.Th = endocortical ost thickness; Ps.O.Th = periosteal ost thickness; Ec.ES/(BS‐OS) = endocortical eroded mineralized bone surface.

^a^ *ρ*, Spearman rank correlation coefficient.

## Results

### Structural and static histomorphometric variables

#### Excess osteoid surface restricts bone turnover in an envelope‐specific manner

**Figure 2:**
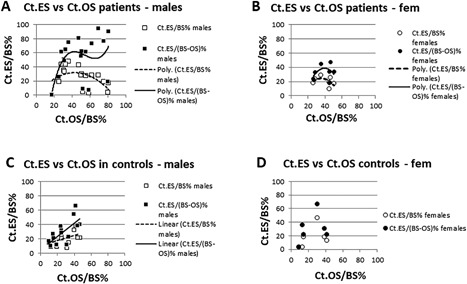
Effects of the extent of cortical osteoid surface (Ct.OS/BS %) on total cortical eroded surface (Ct.ES/BS %) and on erosion of cortical mineralized surface (Ct.ES/(BS–OS) %) in patients and controls of both sexes. (*A*) Male patients with the greatest extent of Ct.OS/BS % had the lowest extent of Ct.ES/BS %. Bone remodeling thus appears to have become uncoupled. However, only erosion of mineralized surface Ct.ES/(BS–OS) % reflects true eroded surface because osteoid cannot be eroded. (*B–D*) Female patients and controls of both sexes had a lesser extent of Ct.OS/BS % and therefore showed less divergence of the two erosion curves than male patients.

Cortical envelope—a remodeling surface: In male and female patients with cortical osteoid surface (Ct.OS/BS %) below 40%, eroded surface (Ct.ES/BS %) and osteoid surface rose in parallel (Fig. 2 *A*), suggesting preservation of coupling of resorption and formation. But where Ct.OS/BS % exceeded 40% in male patients, Ct.ES/BS % was low, bone remodeling appeared to be uncoupled, and Ct.ES/(BS–OS) % values were high (Fig. 2 *A*). In female patients (Fig. 2 *B*), osteoid and eroded surfaces were less extensive, hence the divergence of the two erosion curves Ct.ES.BS % and Ct.ES/(BS–OS) % was minimal. In controls of both sexes (Fig. 2 *C*, *D*), values of Ct.OS/BS % did not exceed 50% and the two erosion curves Ct.ES/BS % and Ct.ES/(BS–OS) % did not diverge.

Endocortical envelope—a modeling surface for net bone loss and gain: In both male and female patients, relationships between endocortical osteoid surface (Ec.OS/BS %) and both Ec.ES/BS % and Ec.ES/(BS–OS) % were similar to those on the cortical envelope. Endocortical control values of both sexes were in similar ranges to cortical control values.

Periosteal envelope—a modeling surface for net bone gain: The ratio of OS/BS % to ES/BS % was highest on the periosteal envelope (Table 2), namely 9.9 on the Ps envelope compared with 1.9 on the Ct envelope and 1.5 on the Ec envelope. Eleven of 24 patients had 100% osteoid cover of the periosteal envelope.

#### Differences between patients and controls were greater in males than in females

In male patients compared with male controls, osteoid surface (OS/BS %) was greater on all three envelopes, and osteoid thickness was greater on the cortical (Ct.O.Th, μm) and the periosteal (Ps.O.Th, μm) envelopes (Table 2). Eroded surface (Ct.ES/BS %, Ec.ES/BS %, Ps.ES/BS %) did not differ between male patients and controls on any envelope. However, eroded surface as % of mineralized bone surface (ES/(BS–OS) %) on the cortical and the endocortical envelopes was significantly higher in male patients than in controls (Table 2).

Lower mineralized bone volume (Md.V/BV %) in male patients than in controls was attributable to the combined effect of greater cortical osteoid volume (Ct.OV/BV %) and marginally greater cortical void volume (Ct.Vd.V/BV %) in the patients (Table 2). The above differences between patients and controls were attributable to osteoid excess in male patients. Females showed no significant differences in any of the histomorphometric variables between patients and controls (Table 2).

#### Differences between male and female patients

**Figure 3:**
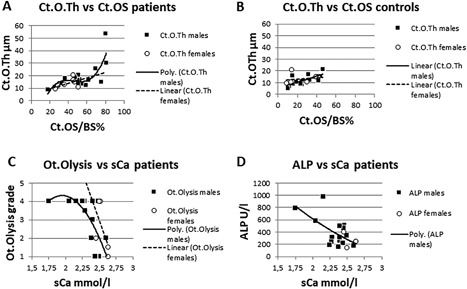
(*A*, *B*) Sex‐specific cortical osteoid thickness (Ct.O.Th) in patients and controls. In male patients, the highest values of Ct.OS/BS were associated with excessive Ct.O.Th. In controls, normal Ct.OS/BS was associated with normal Ct.O.Th. (*C*) In male patients, low sCa levels were associated with increased grades of osteocytic osteolysis (Ot.Olysis) and (*D*) with raised alkaline phosphatase (ALP) levels.

In male patients, values of Ct.OS/BS % and Ec.OS/BS % above 50% were associated with a decline in eroded surfaces Ct.ES/BS % (Fig. 2 *A*) and Ec.ES/BS% but with a divergent steep rise in eroded mineralized surfaces Ct.ES/(BS–OS) % and Ec.ES/(BS–OS)%. Divergence of erosion curves was minimal in female patients (Fig. 2 *B*) and in controls of both sexes (Fig. 2 *C*, *D*) because osteoid surface was less extensive. Furthermore, in male patients, but not in female patients, cortical erosion Ct.ES/BS % correlated with cortical porosity Ct.Vd.V/BV % (male patients *r* = 0.698, *p* < 0.0001; female patients *r* = 0.374, *p* = 0.209). Where cortical osteoid surface Ct.OS/BS % exceeded 60% in male patients, cortical osteoid thickness was abnormally elevated (Fig. 3 *A*). In contrast, in female patients (Fig. 3 *A*) and in controls of both sexes (Fig. 3 *B*), cortical osteoid surface did not exceed 60%; hence, cortical osteoid thickness was only minimally elevated. Hyperosteoidosis was thus more severe in male than in female patients and controls of both sexes.

### Features of demineralization within mineralized cortical bone tissue

#### Osteocytic osteolysis (Ot.Olysis) grades

**Figure 4:**
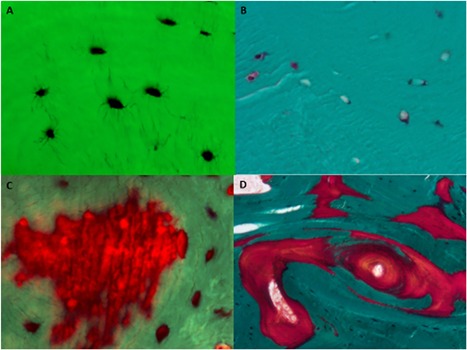
(*A–D*) Photomicrographs. (*A*) Osteocytic osteolysis (Ot.Olysis) grade 3. Osteocyte lacunae stain dark, and dark‐staining tortuous canaliculi pass from lacunar surfaces toward other osteocytes or toward an osteoid surface in a male patient aged 16 years with low sCa (original mag ×400). (*B*) In a control male aged 17 years, canaliculi are invisible (original mag ×400). (*C*) Periosteocytic osteolysis (Peri.Ot.Olysis). In a group of osteocytes, most cells are surrounded by a red halo of demineralized bone (Peri.Ot.Olysis). The group of osteocytes forms a red‐staining “island” of demineralized bone tissue within green‐staining mineralized bone. Male patient aged 15 years with pre‐osteomalacia (original mag ×400). (*D*) Osteomalacia with extensive areas of osteoid of irregular shape (red) throughout cortical bone (green). Male patient aged 16 years (original mag ×100).

Ot.Olysis was found more commonly in patients (*n* = 21/26 [81%], 15 males, 6 females) than in controls (*n* = 7/20 [37%], 4 males, 3 females), *p* = 0.006, chi‐square test), and its histological appearance differed between the two groups. In patients, Ot.Olysis showed numerous long tortuous canaliculi emanating perpendicularly from the lacunar surface in the direction of other lacunae (Fig. 4 *A*) or a nearby osteoid surface. In controls, canaliculi were either not observed (Fig. 4 *B*) or were limited to a few short canaliculi within newly forming osteons presumably undergoing secondary mineralization. In patients, Ot.Olysis correlated negatively with sCa (Table 5, Fig. 3 *C*) and positively with Peri.Ot.Olysis and two osteoid variables (Table 5). In controls, Ot.Olysis grades correlated positively with osteoid volume (Ct.OV/BV% *ρ *= 0.535, *p* = 0.018, Spearman rank correlation coefficient). In the most advanced form of Ot.Olysis (grade 4), lacunae were surrounded, singly or in groups, by a bright red perilacunar area of osteoid of varying shapes and sizes. This feature constituted the transition from Ot.Olysis to Peri.Ot.Olysis (Fig. 4 *C*).

#### Periosteocytic osteolysis (Peri.Ot.Olysis) μm^3^

In 6 of 25 patients (5 males, 1 female) but in none of the controls did mineralized green‐staining bone contain red‐staining, irregularly shaped “islands” of Peri.Ot.Olysis (Fig. 4 *C*). All bone specimens with Peri.Ot.Olysis also displayed Ot.Olysis, but the converse was not the case. The extent of Peri.Ot.Olysis correlated negatively with serum calcium, 25OHD, and mineralized bone volume (Md.V/BV %), and positively with 5 of 7 osteoid variables (Table 5).

### Serum biochemistry

Serum creatinine levels were normal in all patients. Serum calcium levels were lower in male than in female patients (15 males 2.32 ± 0.21 mmol/L; 4 females 2.55 ± 0.09 mmol/L; *p *= 0.024). Patients with the lowest sCa levels (males) had the highest values of ALP (Fig. 3 *D*). Correlations between histomorphometric and serum biochemical variables are given in Table 4.

## Discussion

### Age and sex

The higher prevalence and greater severity of rickets in males than females, particularly between ages 16 and 19 years, may reflect later skeletal maturity in South African black males at age 17 years compared with black females at age 15 years.^16^ Because the average age at initiation of puberty (transition from Tanner stage 1 to Tanner stage 2) was found not to differ significantly between South African black males and females (range 9.8 to 10.5 years^17^), males are expected to experience a longer skeletal growth period, namely from about age 10 to 17 years compared with 10 to 15 years in females.^16^ Additionally, peak height velocity in black males is greater than in black females.^18^ These sex‐specific differences in duration and rate of bone growth may account for the greater number of males being affected by this bone disease and to its greater severity in males during late adolescence.

### Anatomical factors

The predilection of the knee region for growth‐related deformities of rickets in adolescents may be based on anatomical factors. Longitudinal growth of the lower limbs occurs predominantly in the knee region. Approximately two‐thirds of longitudinal growth of the femur takes place at its distal growth plate, and two‐thirds of longitudinal growth of the tibia at its proximal growth plate. These two growth plates are among the last to close and do so around age 20 years in males^19^ and about 3 years earlier in females. The growth plates at the knee are thus at risk of developing rachitic deformities until a later age in males than in females, particularly in the presence of calcium deficiency.

### Biochemistry

Biochemical changes in this condition reflect a disordered bone‐related endocrine environment initiated by declining serum calcium levels causing secondary hyperparathyroidism.^20^ Although the PTH‐induced rise in 1,25(OH)_2_D improves intestinal calcium absorption, it also inhibits osteoid mineralization,^21^, ^22^ thus contributing to hyperosteoidosis. When osteoid surface (Ct. OS/BS%) covered in excess of 50% of bone surface, serum calcium levels progressively declined because the extensive osteoid surface reduced available bone surface that could be used for osteoclastic resorption.

Furthermore, 1,25(OH)_2_D stimulates 24‐hydroxylase activity increasing the catabolism of 25OHD to the inactive compound 24,25(OH)_2_D, thus increasing the risk of developing vitamin D insufficiency with dietary calcium deficiency.^8^, ^23^ Raised PTH levels also lead to renal phosphate loss^24^ and hypophosphatemia that aggravates the bone mineralization defect. Thus, these patients eventually develop a triple insufficiency of calcium, 25OHD, and phosphate, with severe adverse effects on bone mineralization, namely secondary hyperparathyroid bone disease (high bone turnover with hyperosteoidosis), and eventually osteomalacia (low or no bone turnover due to hyperosteoidosis), both causing derangements of remodeling and modeling of bone.^1^, ^8^, ^25^

### Envelope‐specific histomorphometric abnormalities

Cortical bone remodeling by resorption and formation in healthy black adolescents are in balance.^26^ In calcium deficiency, remodeling increased until accumulating osteoid surface (Fig. 2 *A*) reduced the available bone surface for resorption. With the decline in eroded bone surface (ES/BS%), ES/(BS–OS)% became the true eroded surface because osteoid is nonfunctional. This effect of resorption blockade by osteoid appeared to have taken over from normal mechanical strains determining normal cortical bone architecture, which now became increasingly disordered and in the worst cases resembled a wide‐meshed sponge. In 3 of the worst affected males, 1 aged 16 years and 2 aged 19 years, we hypothesize that low serum calcium levels began to fall, and secondary hyperparathyroid bone disease had resulted in osteomalacia.

The normal endocortical envelope during adolescence is a modeling surface characterized by intermittent net bone loss and gain.^14^ We suggest the blockade by osteoid had prevented the erosion required to shape an endocortical surface that distinguishes the more compact cortical from trabecular bone. Furthermore, strains necessary to achieve this distinction were presumably rendered ineffectual by existing architectural distortions in cortical bone. Whereas all control bone samples had a well‐defined cortico‐cancellous border, this was found in only 35% of patients. In the remaining patients, all males aged 15 to 19 years, poor cortico‐cancellous definition was associated with high intracortical porosity that made distinction of an endocortical envelope between cortical and trabecular bone uncertain. This poor distinction may account for poor definition of the cortico‐cancellous junction found on radiographs of diaphyseal bone in rickets.^8^ By inference, this radiological finding in rickets suggests advanced bone disease.

When hyperosteoidosis (OS/BS %) on the cortical and endocortical envelopes rose above 50% to 60%, calcium deficiency appeared to have reached a critical level as indicated by four important changes: 1) sCa levels declined; 2) ALP rose; 3) osteoid thickness rose steeply; and 4) ES/BS % declined, although the more appropriate ES/(BS–OS) % remained elevated. Thus, in the absence of a bone biopsy, a decline in sCa and a rise in sALP in such patients suggest that an advanced degree of calcium deficiency bone disease has been reached that calls for urgent treatment.

The periosteal envelope differed from the other two envelopes in that it was least adversely affected by this bone disease. As a natural modeling surface for bone growth in width, the high OS/BS % to ES/BS % ratio is here beneficial. Indeed, DXA‐derived bone area (cm^2^) in the radial diaphyses of Nigerian children with dietary calcium deficiency rickets was found to be greater than in healthy controls,^27^ a finding also noted in South African children.^28^ Because strength of a bone increases with the fourth power of its radius, this outcome may be the only positive feature of this bone disease—once the periosteal osteoid mineralizes.

### Other features of compromised bone mineralization

#### Osteocytic osteolysis (Ot.Olysis)

In healthy bone, lacunar‐canalicular porosity (LCP) and vascular porosity (VP) are of comparable size, namely LCP 1% to 14% and VP 4% to 16% of bone volume.^29^ In calcium deficiency, both porosities enlarge, VP through the action of osteoclasts and LCP through that of osteocytes.^29^ In this process, osteocytes remove perilacunar and pericanalicular mineral through molecular mechanisms known to be used by osteoclasts during calcium‐demanding states such as lactation.^30^, ^31^ During this process, the LCP enlarges but decreases again on cessation of the period of raised calcium demand. Furthermore, during lactation in mice, a significant decline in bone tissue level elastic modulus and its full recovery post‐weaning mediated by osteocytes have been recorded.^32^ We expect that calcium deficiency rickets may likewise lead to a decline in cortical bone elastic modulus as a result of the abnormal increase in unmineralized bone tissue, namely resorption space, osteoid volume, LCP (Ot.Olysis), and demineralized bone volume of Peri.Ot.Olysis.

The presence of Ot.Olysis within newly forming osteons in control subjects may reflect the physiologically high demand for calcium during the phase of secondary mineralization of recently deposited bone.^33^

#### Periosteocytic osteolysis (Peri.Ot.Olysis): mineralization failure or demineralization?

“Islands” of red‐staining osteoid within green‐staining mineralized bone tissue could denote either mineralization failure after osteoid deposition or demineralization of previously mineralized bone tissue. Because outlines of many osteoid islands did not follow normal microanatomical lines of mineralization along newly deposited collagen fibers but rather cut across them (Fig. 4 *C*, *D*), demineralization was the more likely origin of osteoid islands.

Whatever led to Peri.Ot.Olysis, its pathogenesis was related to calcium deficiency bone disease as indicated by its negative correlations with both Ca and 25(OH)D levels (Table 5). Ot.Olysis correlated negatively with sCa only and that less highly than Peri.Ot.Olysis (Table 5). Also, in Ot.Otlysis, demineralization was confined to the lacunar‐canalicular space, whereas in Peri.Ot.Olysis, demineralization extended beyond these confines. Furthermore, Peri.Ot.Olysis was more highly positively correlated with more osteoid variables than did Ot.Olysis. These findings suggest that Peri.Ot.Olysis represents a more advanced stage of demineralization than does Ot.Olysis. The phenomenon of hypomineralized periosteocytic lesions (HPL) has been previously described in bone biopsies of patients with X‐linked hypophosphatemia in whom serum calcium levels are normal.^34^

Importantly, the findings of Ot.Olysis and Peri.Ot.Olysis show that bone tissue abnormalities of calcium deficiency rickets are not confined to the growth plate and to bone surfaces but also manifest at the level of the osteocyte and its processes and in advanced cases extend into surrounding tissues as Peri.Ot.Olysis. Although belonging to the same cell lineage, osteoblasts and osteocytes appear to react differently to calcium deprivation. Whereas osteoblasts on bone surfaces are associated with mineralization failure in the form of wide seams of newly deposited osteoid, osteocytes and their processes in the depth of bone show evidence of active demineralization of previously mineralized bone tissue and of its remineralization.^31^

It is not known how long restoration of cortical architecture in adolescents with calcium deficiency osteomalacia would take once treatment is commenced. Nor is there information on the long‐term outcome of bone density and bone microstructure in adulthood of individuals who had nutritional rickets during childhood or adolescence, treated or untreated.

### Limitations

The small number of female patients (*n* = 7) rendered results of statistical analysis by sex of questionable value. Serum PTH values in patients would have been helpful in confirming hyperparathyroidism. Because most control bone samples came from necropsies, no past history and no serum biochemical values were available. In the previous trabecular bone study of the present subjects, one patient's sex (case 26) had erroneously been entered as female; this error has been corrected in the present study. The error in the previous study would not have affected results of that study^10^ because no sex‐specific analyses had been carried out.

In summary, cortical bone histomorphometry in black adolescents with calcium deficiency rickets showed bone disease ranging from hyperosteoidosis and histological hyperparathyroidism to osteomalacia. Because osteoid was neither being mineralized nor eroded, it progressively blocked mineralized bone surface needed for bone remodeling and to release calcium. Serum Ca and 25(OH)D declined, and 1,25(OH)_2_D and ALP rose. Moreover, two features suggesting osteocyte‐mediated demineralization of cortical bone were observed: 1) osteocytic osteolysis within the lacunar‐canalicular space, and 2) islands of periosteocytic osteolysis within mineralized bone. Thus, skeletal pathology of calcium deficiency rickets was not confined to the growth plate and to bone surfaces but was also evident at osteocyte level.

## Disclosures

The authors state that they have no conflicts of interest.
